# A Wire Bow Model of Diamond Wire Sawing with Asymmetric Arc Hypothesis

**DOI:** 10.3390/mi14051004

**Published:** 2023-05-06

**Authors:** Zhikui Dong, Chenpu Zhang, Ziliang Liu, Yanheng Zhao, Ke Xing, Wenming Guo

**Affiliations:** 1 School of Mechanical Engineering, Yanshan University, No. 438 West Hebei Avenue, Qinhuangdao 066004, China; 2 Plant 4, Fuyang Industrial Building, Fuyuan Road, Xiangcheng District, Suzhou 215100, China; 3 XCMG Earthmoving Machinery Division, NO. 99 Kunpeng Bei Road, Xuzhou Economic Development Zone, Xuzhou 221004, China

**Keywords:** diamond wire sawing, asymmetric arc hypothesis, endpoint tension, cutting force, wire bow deflection

## Abstract

Diamond wire sawing is the main processing method for hard and brittle materials, but the unreasonable matching of process parameters will reduce its cutting ability and stability. In this paper, the asymmetric arc hypothesis of a wire bow model is proposed. Based on this hypothesis, an analytical model of the wire bow between the process parameters and the wire bow parameters was established and verified with a single-wire cutting experiment. The model considers the asymmetry of the wire bow in diamond wire sawing. The tension at both ends of the wire bow is called the endpoint tension; by calculating the difference in tension between the two ends, a reference for the cutting stability and a tension range for the selection of the diamond wire were provided. The model was used to calculate the wire bow deflection and the cutting force, providing theoretical guidance for the matching of process parameters. Based on the theoretical analysis of the cutting force, endpoint tension and wire bow deflection, the cutting ability, cutting stability, and the risk of wire cutting were predicted.

## 1. Introduction

Currently, wire sawing technology is the main processing technology for cutting hard and brittle material. Sliced materials include sapphire, monocrystalline silicon, polycrystalline silicon, rare-earth permanent-magnet materials, etc., which are widely used in many industries. As this is the first process for machining hard and brittle ingots, the efficiency and quality of wire sawing have a direct influence on the subsequent processes. Therefore, it is increasingly important to systematically study the wire sawing principle for hard and brittle materials.

Wire sawing technology is mainly divided into two categories according to the abrasive state. One is free abrasive wire sawing, but the cutting efficiency of this method is relatively low and the utilization rate of abrasives is relatively insufficient. The other is fixed abrasive wire sawing. Diamond wire sawing is a kind of fixed abrasive wire sawing that has the advantages of having a strong abrasive holding force and high cutting efficiency, making it the mainstream sawing technology utilized in industry.

Through experimental research on how the properties of diamond wire and process parameters affect the cutting force, scholars have found that the cutting quality can be judged by the cutting force. Clark, Hardin, and Ge PQ [1,2,3] found that increasing the size of diamond abrasive particles, increasing the wire speed, and reducing the feed speed will reduce the normal and tangential cutting force and then will reduce the crack damage on the surface and improve the cutting quality. Based on these results, Pala U [4] found that increasing the wire speed will reduce the cutting force and further improve the surface finish, as well as cause the change rate of the cutting force to gradually decrease. The cutting force of a single abrasive particle determines the cutting depth and affects the material-removal mode and crack propagation length; thus, the cutting force is the key factor affecting the surface quality of wafers [5].

Due to the obvious effect of cutting force on cutting quality, some scholars have begun to establish a micromechanical model of the interaction between diamond abrasive particles and ingots to study the cutting force. Wang [6] established a theoretical force model for the nano- and microscratching of a single diamond-abrasive particle in the wire sawing of silicon carbide (SiC). Ge MR [7] analyzed the distribution and change in the sawing stress field during the slicing of potassium dihydrogen phosphate (KDP) crystal and explored the effects of cutting force concentration on cutting quality. Li ZQ [8] considered the anisotropy of silicon and established a cutting force model to obtain the surface shape deviation of silicon. Wang PZ [5] proposed a calculation method to determine the cutting force of a single abrasive particle considering the effect of friction and lateral cracks on the material removal and obtained a cutting force model based on process parameters and wire parameters.

Subsequent scholars extended the cutting force model of a single abrasive particle to the macro level of diamond wire sawing. Wu CH [9] analyzed the force of a single diamond-abrasive particle, obtained the calculation formulas of the normal and tangential cutting force, and established the macro mathematical model between the cutting force and the three process parameters of wire speed, feed speed, and wire radius. Li SJ [10,11] established the macro cutting force model based on wire speed, feed speed, contact length, and cutting force. Based on this model, Tang AF [12] established a macro cutting force model by using a finite element simulation and analyzed the effects of feed speed and wire speed on cutting force.

When constructing the cutting force model, most of the above scholars assumed the shape of the diamond wire in the cutting process to be a straight line, ignoring the bending deformation of the diamond wire, which is called the wire bow. The wire bow is an important factor that affects cutting ability and machining accuracy [13]. The state of the wire bow reflects the difficulty of cutting ingots and the quality of cutting and thus plays an important role in the research of diamond wire sawing. Therefore, some scholars have studied the effect of the wire bow on the sawing process through experiments and theoretical derivations. Clark [14] first proposed a method to convert wire bow deflection into a vertical cutting force. Kim, D [15] showed that increasing the wire bow deflection in multi-wire sawing will increase the cutting load, which will lead to severe wear of the wire and affect the cutting quality. Liu, TY [16] proposed the rationality of using the wire bow angle to describe the process parameters. Qiu, J [17] proposed that increasing the wire bow causes the cutting ability of diamond wire to decrease gradually. Lin, ZS [18] established the simulation model of wire bow deflection and determined that the wire bow can be described as a quadratic curve. Liedke [19] established an analytical model of the free abrasive wire sawing process, linking the process parameters with the shape and cutting force of the wire bow, but the model is only applicable to free abrasive wire sawing and ignores the effect of the tangential cutting force on the shape of the wire bow.

The above scholars have begun to explore the effect of process parameters on the de-flection and deflection angle of the wire bow and found that the bending degree of the wire bow has a significant influence on the cutting quality and efficiency, but no mathematical model of the fixed abrasive wire bow with the process parameters has been established. There is no systematic research on the relationship between the process parameters and wire bow parameters, where the process parameters include wire speed, feed speed, wire tension, wire diameter, wire length, and contact length, and the wire bow parameters include cutting force, wire bow deflection, deflection angle, and endpoint tension at both ends of the wire bow.

In the process of diamond wire sawing, the excessive bending deformation and cutting force of the wire saw reflect the mismatch of process parameters such as wire speed, feed speed, and wire diameter, which in turn reflects the insufficient cutting ability of the wire saw, that is, the inability to effectively remove materials. Similarly, excessive deformation of the wire saw can reduce its stability during the cutting process, making it prone to defects such as slice bending, an increase in surface roughness, and surface fragmentation and distortion during wire sawing. Therefore, modeling and analyzing the bending degree of wire saws can achieve accurate calculations of wire bow deflection, wire saw tension changes, and cutting forces, which can reduce the time required for wire bow suppression, and then theoretically analyze and predict the cutting ability and stability of the diamond wire sawing.

In this study, the bending deformation of the diamond wire was considered, the displacement deformation of the wire bow was calculated and theoretically combined with the results of the finite element calculation, and the asymmetric arc hypothesis of the wire bow deformation was proposed. By analyzing the force of diamond abrasive particles when removing material, and by considering the distribution of abrasive particles on the wire saw and the deformation of the wire saw, the cutting force of the wire saw can be obtained theoretically. An analytical model of the asymmetric wire bow between process parameters and wire bow parameters that is suitable for fixed abrasive wire sawing is established, and experiments are designed to verify it. The wire bow model describes the cutting forces, the deformation of the wire saw and the tension change in the wire saw with different parameters at the macro level.

## 2. Materials and Methods

### 2.1. Construction Method of Wire Bow Model

**Figure 1:**
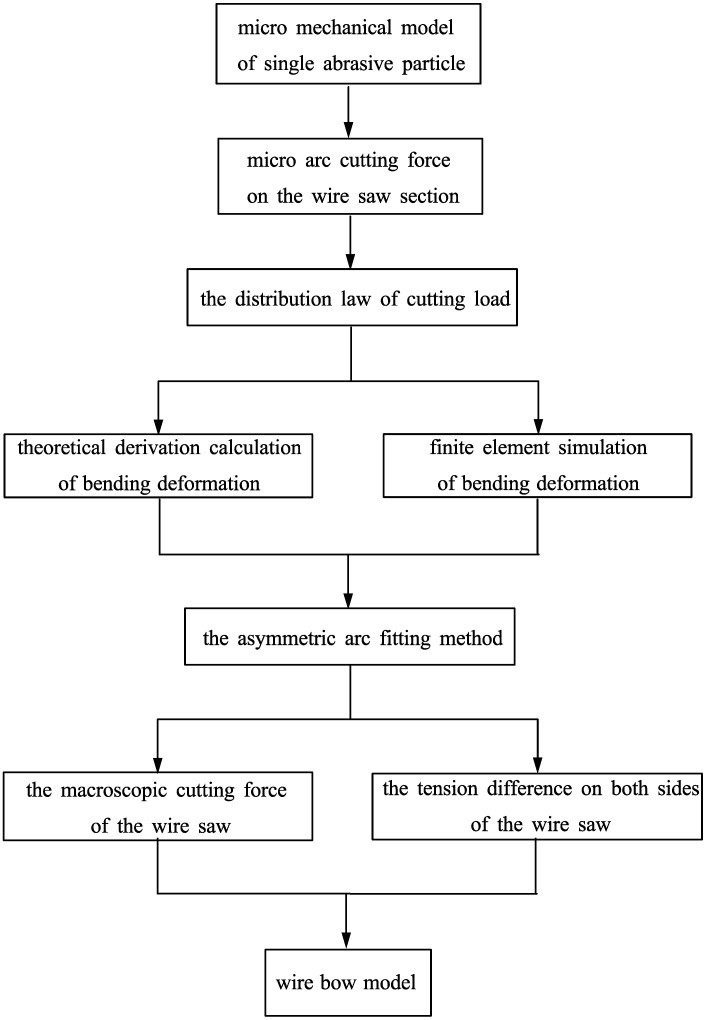
The mind map for constructing the wire bow model.

This article first establishes a micro mechanical model of a single abrasive particle and extends it to the calculation of micro-arc cutting force on the wire saw section. On this basis, the distribution law of cutting load on the wire saw is determined. Based on the load distribution law of the cutting segment, finite element simulation and theoretical derivation calculation were conducted on the bending deformation of the wire saw. By comparing and analyzing the simulation results with the numerical fitting results, the reliability of the asymmetric arc fitting method was determined. Considering the bending deformation of the wire saw, combined with the arc fitting method, the macroscopic cutting force of the wire saw was theoretically derived and calculated, and the tension difference on both sides of the wire saw was analyzed, thus completing the construction of the wire bow model. Subsequently, the accuracy of the analytical model was verified by comparing the calculated results of the model with the experimental results, and the regularity was explored through experiments. A mind map constructed by the wire bow model is shown in Figure 1.

### 2.2. Calculation of Micro-Arc Cutting Force on Cutting Segment of Wire Bow

**Figure 2:**
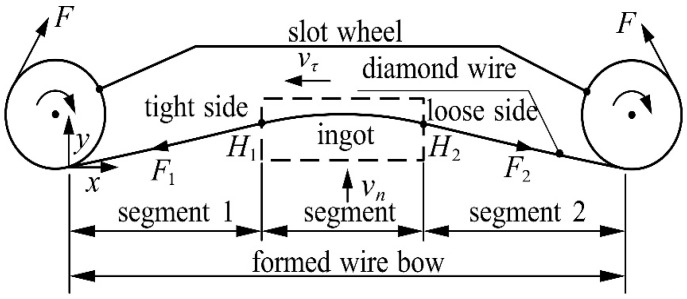
Schematic diagram of the wire bow.

The diamond wire is subjected to the cutting force generated by the interaction between the ingot on the lifting table and the diamond abrasive particle, which causes the diamond wire to be deformed. The bending deformation of diamond wire is called the wire bow. Taking the wire speed to the left as an example, Figure 2 is a schematic diagram of the wire bow. The diamond wire affected by the cutting force is divided into three parts, including the cutting segment in the middle and the non-cutting segments on both sides. Due to the tangential cutting force, the tension on both sides of the cutting section is different, resulting in a loose edge on one side and a tight edge on the other. F is the diamond wire tension (N); $F_{1}$ is the tension at endpoint $H_{1}$ of segment 1 (N), which is also called the tension on the tight side; and $F_{2}$ is the tension at endpoint $H_{2}$ of segment 2 (N), which is also called the tension on the loose side. Asymmetric arcs result from the existence of a loose side and tight side. $F_{1}$ and $F_{2}$ are collectively referred to as the endpoint tension, $v_{n}$ is the feed speed of the ingot (mm/h), and $v_{\tau }$ is the diamond wire speed (m/s). Taking the end of non-cutting segment 1 as the origin, the displacement of the diamond wire in the y direction is called the wire bow deflection (mm).

The abrasive particle is simplified as a conical rigid body when calculating the micro cutting force. The morphology of abrasive particles is approximately hexahedral, octahedral, deformed, etc. According to the research on the depth of abrasive cutting, the top of the abrasive is the part that acts on the workpiece material when cutting. To simplify the analysis, the shape of the top of the abrasive particle is considered equivalent to a cone. Considering the distribution law of abrasive particles on a wire saw, the abrasive particle distribution on a line saw was approximated as a uniform distribution.

**Figure 3:**
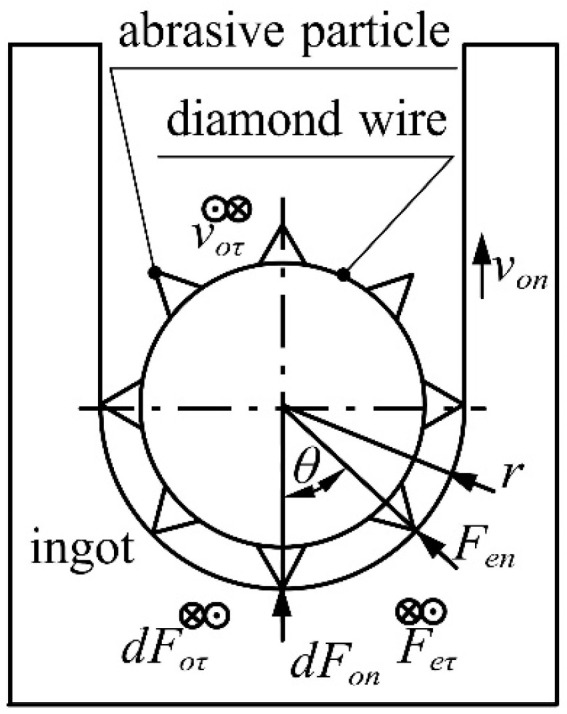
Simplified force model of diamond wire section.

The essence of a diamond wire is a metal wire with consolidated diamond-abrasive particles; taking the cross-section of a diamond wire for analysis, with a simplified model shown in Figure 3, the normal force of the cross-section is the resultant force of all abrasive particles participating in cutting along the direction of the feed speed, and the tangential force of the cross-section is the resultant force of all abrasive particles participating in the cutting along the direction of the wire speed. $v_{o\tau }$ is the wire speed perpendicular to the cross-section direction (m/s); $v_{on}$ is the feed speed parallel to the vertical direction of the cross-section (mm/h); θ is the angle of the circumferential position of the abrasive particle on the diamond wire cross-section (°); r is the diamond wire radius (mm); $F_{en}$ is the normal cutting force of the abrasive particle (N); $F_{e\tau }$ is the tangential cutting force of the abrasive particle (N); and $dF_{on}$ is the cutting force parallel to the vertical direction of the cross-section (N), that is, the normal cutting force (N) on the micro-arc of the cutting segment. $dF_{o\tau }$ is the cutting force perpendicular to the cross-section (N); that is, it is the tangential cutting force on the micro-arc of the cutting segment (N).

The force of a single diamond abrasive particle on the diamond wire when removing the material [10] is

(1) $$\left\{ \begin{array}{l} F_{en}=\left(K\tan\beta +\pi \sigma_{sy}\tan^{2}\beta \right)h_{\theta }{}^{2}\\ F_{e\tau }=\left(\frac{K\pi }{4}+\mu \pi \sigma_{sy}\tan^{2}\beta \right)h_{\theta }{}^{2} \end{array}\right.$$

In the formula, $F_{en}$ is the normal cutting force of the abrasive particle (N), $F_{e\tau }$ is the tangential cutting force of the abrasive particle (N), K is the specific cutting force of the normal cutting force (N/mm^2^), β is the cone half angle of the abrasive particle (°), μ is the friction coefficient between the abrasive particle and the ingot, and $h_{\theta }$ is the average thickness of the undeformed chips at the circumferential angle θ of the diamond wire (mm). $\sigma_{sy}$ is the average contact pressure strength between the wear plane of the abrasive particle and the ingot (MPa).

Assuming a uniform shape of abrasive particles distributed evenly on the wire, we can consider the bending of the wire as the summation of the normal and tangential cutting force of all abrasive particles that are involved in the cutting on the cross section:

(2) $$\left\{ \begin{array}{l} dF_{on}=2\int_{0}^{\frac{\pi }{2}}F_{en}\cos\theta nrd\theta dS\\ dF_{o\tau }=2\int_{0}^{\frac{\pi }{2}}F_{e\tau }nrd\theta dS \end{array}\right.$$

In the formula, n is the number of abrasive particles per unit area (1/mm^2^) and dS is the micro-arc length of the diamond wire (mm).

**Figure 4:**
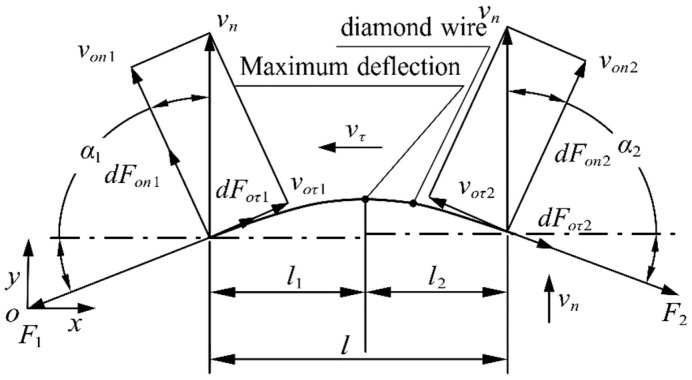
Force model of wire bow.

Extending the force of the diamond wire section to the micro-arc segment, as shown in Figure 4, creates a force model of the wire bow. Due to the bending of the cutting segment, the normal and tangential velocities of the cross-section vary with the angle of wire bending, and the cutting segment is divided into two segments at the point of maximum deflection for analysis. l is the contact length (mm), where the feed speed of segment $l_{1}$ will attenuate the tangential speed of its section, and the feed speed of segment $l_{2}$ will promote the tangential speed of its section. Due to the existence of the tangential velocity, the cutting part is divided into a loose side and tight side, which also causes the wire deflection to be asymmetric. $\alpha_{1}$ is the angle between the diamond wire and the *x*-axis (°), which is the deflection angle of the tight side, and $\alpha_{2}$ is the deflection angle of the loose side.

The cutting depth of the diamond abrasive particle is

(3) $$h_{\theta }=\sqrt{\frac{v_{on}\cos\theta }{nv_{o\tau }\tan\beta }}$$

The normal and tangential velocities of the cross-section vary with the bending angle of the wire bending, and the average thickness of the undeformed chips at the θ of the cross-section corresponding to $l_{1}$ and $l_{2}$ is

(4) $$\left\{ \begin{array}{l} h_{\theta 1}=\sqrt{\frac{v_{on1}\cos\theta }{n\left(v_{\tau }-v_{o\tau 1}\right)\tan\beta }}=\sqrt{\frac{v_{n}\cos\alpha_{1}\cos\theta }{n\left(v_{\tau }-v_{n}\sin\alpha_{1}\right)\tan\beta }}\\ h_{\theta 2}=\sqrt{\frac{v_{on2}\cos\theta }{n\left(v_{\tau }+v_{o\tau 2}\right)\tan\beta }}=\sqrt{\frac{v_{n}\cos\alpha_{2}\cos\theta }{n\left(v_{\tau }+v_{n}\sin\alpha_{2}\right)\tan\beta }} \end{array}\right.$$

The cutting forces of the two micro-arcs of the diamond wire at two ends are

(5) $$\left\{ \begin{array}{l} dF_{on1}=\frac{\pi rK_{1}}{2}\cdot \frac{v_{n}\cos\alpha_{1}}{v_{\tau }-v_{n}\sin\alpha_{1}}dS\\ dF_{o\tau 1}=2\pi rK_{2}\cdot \frac{v_{n}\cos\alpha_{1}}{v_{\tau }-v_{n}\sin\alpha_{1}}dS \end{array}\right.$$

(6) $$\left\{ \begin{array}{l} dF_{on2}=\frac{\pi rK_{1}}{2}\cdot \frac{v_{n}\cos\alpha_{2}}{v_{\tau }+v_{n}\sin\alpha_{2}}dS\\ dF_{o\tau 2}=2\pi rK_{2}\cdot \frac{v_{n}\cos\alpha_{2}}{v_{\tau }+v_{n}\sin\alpha_{2}}dS \end{array}\right.$$

The comprehensive influence coefficients are calculated as follows:

(7) $$\left\{ \begin{array}{l} K_{1}=K+\pi \sigma_{sy}\tan\beta \\ K_{2}=\frac{K}{4\tan\beta }+\mu \sigma_{sy}\tan\beta \end{array}\right.$$

In the formula, $K_{1}$ is the normal comprehensive influence coefficient (MPa) and $K_{2}$ is the tangential comprehensive influence coefficient (MPa).

Through industrial investigation, it can be found that the turning angles of the cutting segments $l_{1}$ and $l_{2}$ in the wire-cutting process are within 20°; the feed speed of the ingot $v_{n}$ is relatively slow, generally not exceeding 100 mm/h; and the wire speed $v_{\tau }$ during normal cutting is relatively fast, being 10–25 m/s. We can substitute $\alpha_{1}$, $\alpha_{2}$, $v_{n}$ and $v_{\tau }$ into the parameter range of Equations (5) and (6):

(8) $$\{\begin{array}{c} dF_{on1}\approx dF_{on2}\\ dF_{o\tau 1}\approx dF_{o\tau 2} \end{array}$$

Then, the cutting force on the cutting segment of the wire bow can be regarded as the loads of equal magnitude in different directions, and its magnitude does not change with the position of the diamond wire.

### 2.3. Asymmetric Arc Hypothesis and Error Analysis

**Figure 5:**
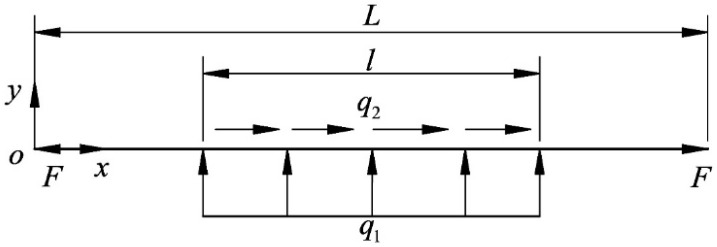
Schematic diagram of the wire bow boundary.

Using a finite element to explore the bending deformation of the wire bow, we can determine the boundary conditions for the finite element calculation of the wire bow according to Equation (8), analyze the working conditions when the wire speed is to the left, and set the wire tension and cutting force when the wire bow is in a stable state. Figure 5 is a schematic diagram of the wire bow boundary, where F is the wire tension (N), L is the wire length (mm), $q_{1}$ is the normal uniform load of the cutting segment (N/mm), and $q_{2}$ is the tangential uniform load of the cutting segment (N/mm).

The finite element simulation is used for simulation, and the diamond wire is simplified into a line body due to the action of tension. In order to always keep the direction of the wire tension along the axis of the wire, a preload is applied to the cutting segment; the two ends of the diamond wire are provided with hinge supports, and a uniform load in the tangential and normal directions is applied.

**Figure 6:**
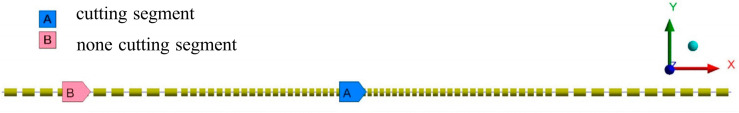
Diagram of wire bow grid division.

**Figure 7:**
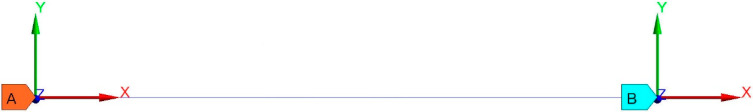
Diagram of hinge support for wire bow.

**Figure 8:**
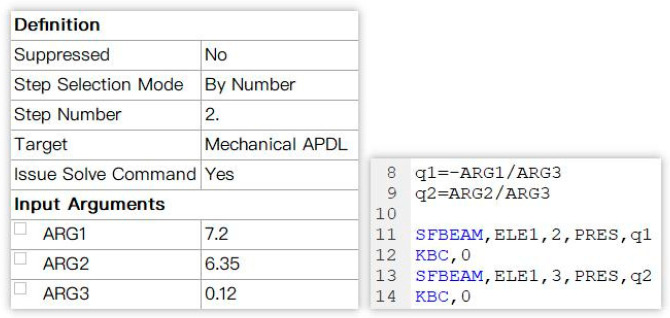
Settings for SFBEAM.

The specific operation settings of the finite element are as follows:

(1) The transient structure module of the finite element software is used for simulation. The diamond wire is simplified into a line body, that is, a beam element model with a circular section. Mesh generation occurs with dense cutting segments and sparse non-cutting segments. Figure 5 shows the grid division diagram of the wire bow. In Figure 6, the middle part is the cutting section with dense grid segments, while the parts near both ends are non-cutting sections with a relatively sparse grid.

(2) The diamond wire is always taut due to the action of tension. Directly applying the force command to both ends of the wire to achieve the effect of tension will lead to a change in the original wire length and cutting segment length. At the same time, this is due to the large displacement generated by this analysis. Thus, to not change the modeling length and to achieve constant tension, the Bolt Pretension command is applied to the cutting segment to keep the tension along the axis.
(3) The two ends of the diamond wire are provided with hinge supports to release the freedom of the z-direction rotation only. Figure 7 shows the hinge support of the wire bow.

(4) The cutting segment needs to exert the tangential and normal uniform load, in combination with commands using the SFBEAM, by changing the input parameters and commands to modify variables, while at the same time facilitating subsequent parameters as shown in Figure 8.

**Figure 9:**
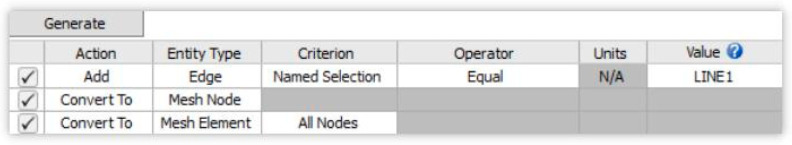
The selections and settings of the wire bow unit.

Named selections are required for establishing the load in commands. Since the SFBEAM object is a unit, the Worksheet command needs to be used to extract the unit corresponding to the selected line body. Figure 9 shows the selections and settings of the wire bow unit.

**Figure 10:**
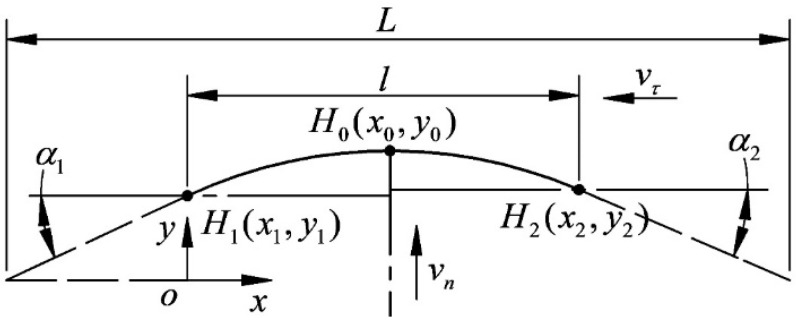
Three-point schematic diagram of the cutting segment.

Figure 10 is a three-point schematic diagram of the cutting segment. According to the finite element calculation results, the coordinate values of the three points of the wire bow are obtained, and the general equation of the circle is used for fitting.

The cutting periods of $H_{1}$, $H_{2}$ and $H_{0}$ coordinates are extracted as follows:

(9) $$\left\{ \begin{array}{l} H_{0}(x_{0},y_{0})\\ H_{1}(x_{1},y_{1})\\ H_{2}(x_{2},y_{2}) \end{array}\right.$$

The expression of the arc passing through the three points is obtained as follows:

(10) $$x^{2}+y^{2}+Dx+Ey+F=0$$

The coefficients *D*, *E*, and *F* are

(11) $$\left\{ \begin{array}{l} D=\frac{\left(l^{2}+y_{2}^{2})(y_{1}-y_{0})+(x_{0}^{2}+y_{0}^{2})(y_{2}-y_{1})+y_{1}^{2}(y_{0}-y_{2}\right)}{l(y_{0}-y_{1})+x_{0}(y_{1}-y_{2})}\\ E=\frac{x_{0}(y_{2}^{2}-y_{1}^{2}+l^{2})+l(y_{1}^{2}-y_{0}^{2}-x_{0}^{2})}{l(y_{0}-y_{1})+x_{0}(y_{1}-y_{2})}\\ F=\frac{y_{1}\left[x_{0}(y_{1}y_{2}-y_{2}^{2}-l^{2})+l(x_{0}^{2}+y_{0}^{2}-y_{0}y_{1})\right]}{l(y_{0}-y_{1})+x_{0}(y_{1}-y_{2})} \end{array}\right.$$

Sensitivity analysis is used to explore which parameters affect the ordinate of three points on the arc, that is, the relationship between three-point deflection and input parameters, so as to simplify the boundary conditions for theoretical derivation of three-point deflection.

**Figure 11:**
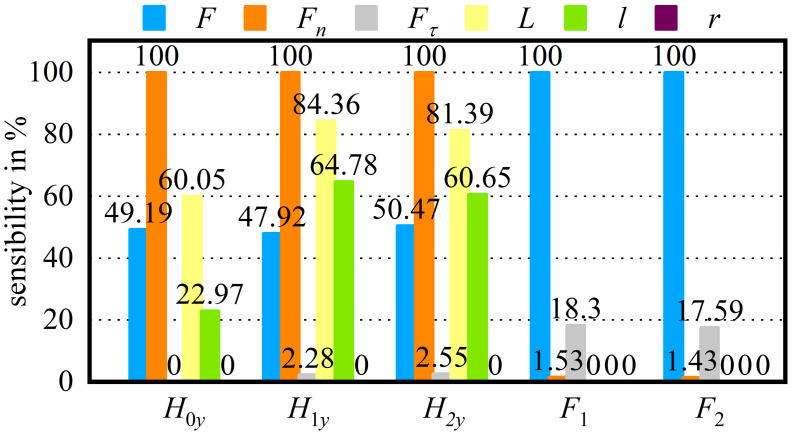
Sensitivity of input variables to output variables.

Finite element simulation is used to set parameters, the range of parameters is set according to Table 1, and 120 test points are generated for calculation. Figure 11 shows the sensitivity of input variables to output variables. $H_{0y}$, $H_{1y}$, $H_{2y}$ are the deflections of $H_{0}$, $H_{1}$, $H_{2}$ (mm).

According to the calculation results,

- $H_{0y}$ is hardly affected by $F_{\tau }$ and r.
- $F_{\tau }$ will affect $H_{1y}$ and $H_{2y}$, which cannot be ignored.
- The effect of r on $H_{1y}$ and $H_{2y}$ is relatively small.
- $F_{1}$ and $F_{2}$ are mainly changed with the F and $F_{\tau }$.

**Figure 12:**
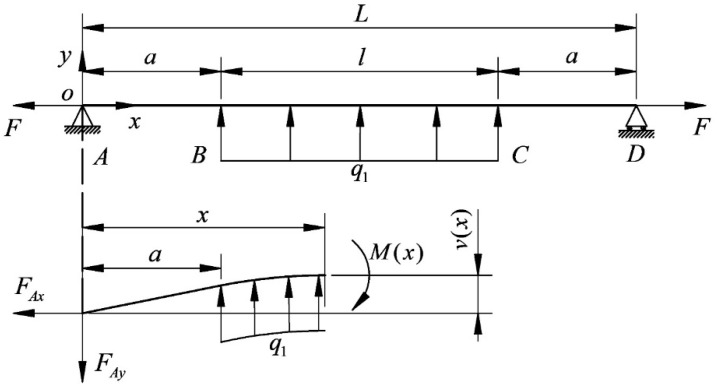
Beam bending model calculated by maximum deflection theory.

It can be seen from the above that the deflection of $H_{0}$ is hardly affected by $F_{\tau }$, so the tangential cutting force can be removed, and the wire bow is simplified as an elastic beam hinged at both ends. Figure 12 shows the beam bending model calculated using maximum deflection theory, and the segment BC is cut for analysis. As the figure shows, $F_{Ax}$ and $F_{Ay}$ are the reaction force at point A (N), α is the distance from the spindle axis to the proximal end of the ingot (mm), M(x) is the moment at x on the segment BC, and v(x) is the deflection at x on the segment BC.

Since the ingot is located in the middle of the main shaft, the endpoint of maximum deflection is at $x=\frac{L}{2}$, and the maximum deflection is obtained as follows:

(12) $$H_{0y}=v{\left(x\right)}_{\max}=v(\frac{L}{2})=\frac{F_{n}\left(2FLl-Fl^{2}-2\pi r^{4}E\right)}{8F^{2}l}$$

In the formula, $H_{0y}$ is the value of maximum deflection (mm), and E is the elastic modulus of diamond wire (MPa).

From Formula (12), it can be seen that the relationship between $H_{0y}$ and $F_{n}$, F, L, and l is relatively large, and since r is relatively small, resulting in a small effect of r on $H_{0y}$, which is consistent with the above sensitivity calculation results.

**Figure 13:**
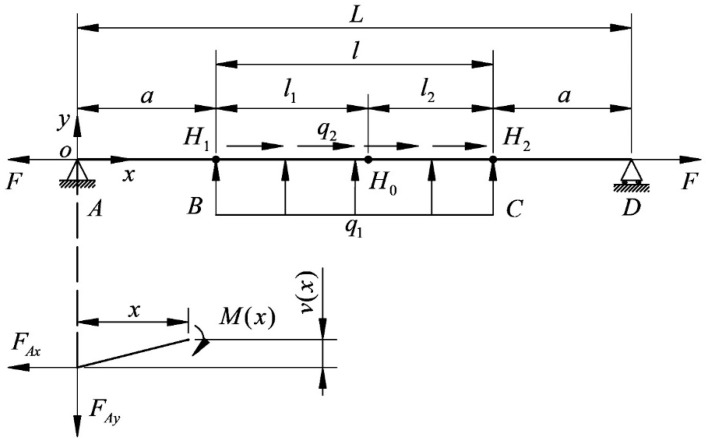
Beam bending model for theoretical calculation of maximum deflection.

According to the results of sensitivity analysis, $H_{1y}$ and $H_{2y}$ are related to the cutting force $F_{n}$ and $F_{\tau }$, and the boundary conditions are retained. Figure 13 shows the beam bending model used for the theoretical calculation of deflection at endpoint $H_{1}$. The segment *AB* is cut for analysis, M(x) is the moment at *x* on the segment *AB* (N⋅mm), and v(x) is the deflection at *x* on the segment *AB*.

The deflection of three points on the bow is determined via theoretical derivation and calculation, and the maximum deflection $H_{1y}$ and $H_{2y}$ are obtained as:

(13) $$\left\{ \begin{array}{l} H_{1y}=v\left(a\right)=\frac{F_{n}\left(L-l\right)}{4\left(F+\frac{F_{\tau }}{l}l_{1}\right)}\\ H_{2y}=v\left(a\right)=\frac{F_{n}\left(L-l\right)}{4\left(F-F_{\tau }+\frac{F_{\tau }}{l}l_{1}\right)} \end{array}\right.$$

In the formula, $H_{1y}$ is the value of deflection at the endpoint $H_{1}$ on the tight side of the wire bow (mm), and $H_{2y}$ is the value of deflection at the endpoint $H_{2}$ on the loose side of the wire bow (mm).

We compare the ordinate of the arc calculated by fitting with the deflection calculated by the finite element and observe the value and law of error corresponding to the various process parameters. According to the investigation, the range of process parameters is presented in Table 1.

The deflection of the fitted arc is compared with the deflection calculated using the finite element to verify the accuracy of the asymmetric arc hypothesis, and the error of the process parameters on the asymmetric arc hypothesis is determined.

**Figure 14:**
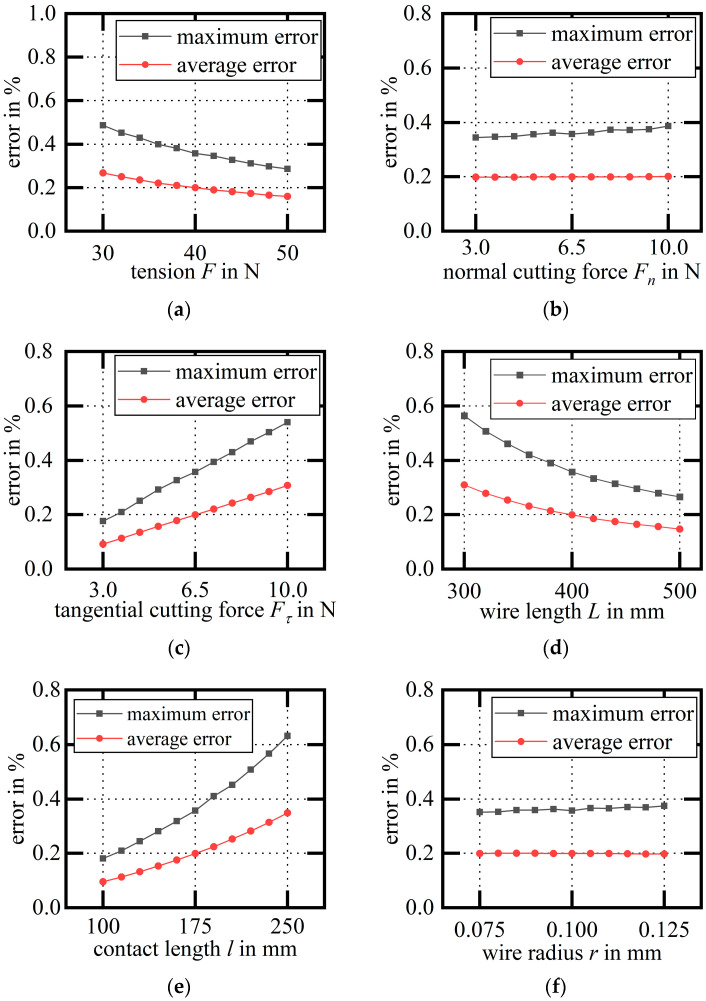
Error diagram of fitting arc for each variable: (**a**) variable F; (**b**) variable $F_{n}$; (**c**) variable $F_{\tau }$; (**d**) variable L; (**e**) variable l; (**f**) variable r.

We can calculate the working-condition parameters corresponding to each variable using the finite element; extract the deflection of the three points $H_{1}$, $H_{2}$, and $H_{0}$ on the cutting segment of the wire bow; and fit the three-point arc. Then, the deflections of each point on the cutting segment calculated using the finite element can be compared with the deflection on the fitted arc, and the maximum relative error and the average relative error of the wire bow can be obtained as shown in Figure 14.

- When F is used as a variable, as the wire tension F increases, the fitting error gradually decreases.
- When $F_{n}$ is used as a variable, the fitting error increases slightly with the increase in the normal cutting $F_{n}$ acting on the cutting segment.
- When $F_{\tau }$ is used as a variable, as the tangential cutting force $F_{\tau }$ acting on the cutting segment increases, the fitting error increases gradually.
- When L is used as a variable, as the wire length L increases, the fitting error gradually decreases.
- When l is used as a variable, as the contact length l increases, the fitting error gradually increases.
- When r is used as a variable, the fitting error increases slightly with the increase in the diamond wire radius r.

According to the changing law of the error diagram, it can be found that the relationship is monotonic, and the variable corresponding to the maximum fitting error within the range of the working condition parameters can be obtained, as shown in Table 2.

**Figure 15:**
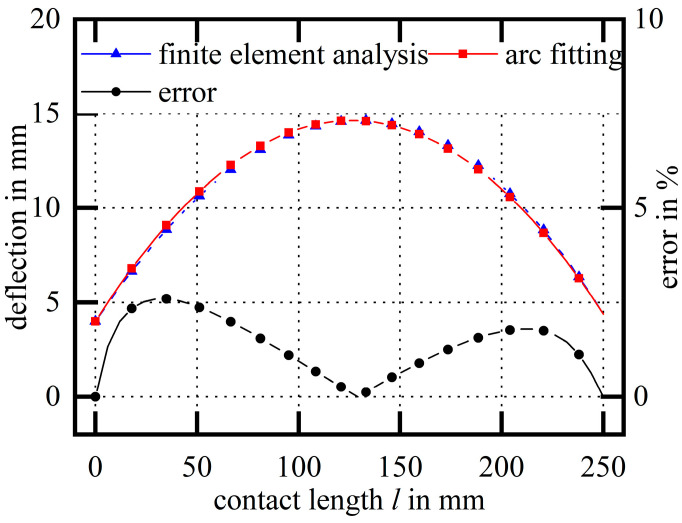
The maximum error diagram of the fitted arc within the working condition.

The working-condition parameters of this table are used as boundary conditions and parameters of the model, and by substituting the parameters into the finite element for calculation, Figure 15 can be obtained as the maximum error diagram of the fitted arc within the range. The deflection calculated by the finite element almost coincides with the deflection of the fitted arc, and the maximum relative error is 2.59%.

Based on the above analysis, and within the range of commonly used working condition parameters, it can be found that the maximum relative error of the deflection between the arc hypothesis and the original curve is small, which shows that the shape of the wire bow can be assumed to be an asymmetric arc.

## 3. Establishment of the Theoretical Wire Bow Model

### 3.1. Establishment of Wire Bow Model

**Figure 16:**
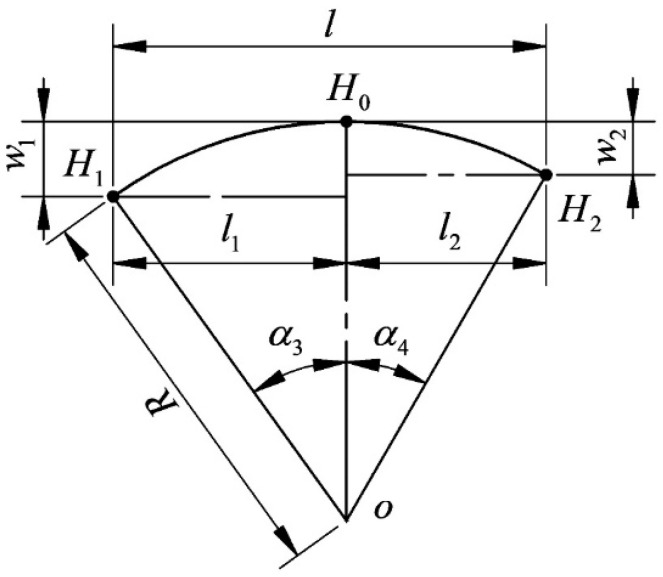
Schematic arc diagram of the cutting segment.

As shown in Figure 16, the cutting segment of the wire bow is assumed to be a three-point arc, and the wire bow model is constructed based on the arc hypothesis. Figure 16 shows a schematic diagram of the cutting segment on the wire bow. $w_{1}$ is the difference in deflection on the tight side (mm), $w_{2}$ is the difference of deflection on the loose side (mm), R is the radius of the fitting arc (mm), $\alpha_{3}$ is the angle of the tight side (°), and $\alpha_{4}$ is the angle of the loose side (°).

According to the geometric properties,

(14) $$\left\{ \begin{array}{l} R=\frac{l^{2}\left(w_{1}+w_{2}\right)-2l\sqrt{w_{1}w_{2}\left(l^{2}+{\left(w_{1}-w_{2}\right)}^{2}\right)}}{2{\left(w_{1}-w_{2}\right)}^{2}}+\frac{w_{1}+w_{2}}{2}\\ \alpha_{3}=\cos^{-1}\left(\frac{l^{2}\left(w_{1}+w_{2}\right)-2l\sqrt{w_{1}w_{2}\left(l^{2}+{\left(w_{1}-w_{2}\right)}^{2}\right)}-{\left(w_{1}-w_{2}\right)}^{3}}{l^{2}\left(w_{1}+w_{2}\right)-2l\sqrt{w_{1}w_{2}\left(l^{2}+{\left(w_{1}-w_{2}\right)}^{2}\right)}+\left(w_{1}+w_{2}\right){\left(w_{1}-w_{2}\right)}^{2}}\right)\\ \alpha_{4}=2\tan^{-1}\left(\frac{lw_{2}+\sqrt{w_{1}w_{2}\left(l^{2}+{\left(w_{1}-w_{2}\right)}^{2}\right)}}{l^{2}+w_{1}{}^{2}-w_{1}w_{2}}\right) \end{array}\right.$$

The difference in deflection can be expressed as

(15) $$\left\{ \begin{array}{l} w_{1}=\frac{F_{n}\left(2FLl-Fl^{2}-2\pi r^{4}E\right)}{8F^{2}l}-\frac{F_{n}\left(L-l\right)}{4\left(F+\frac{F_{\tau }}{l}R\sin\alpha_{3}\right)}\\ w_{2}=\frac{F_{n}\left(2FLl-Fl^{2}-2\pi r^{4}E\right)}{8F^{2}l}-\frac{F_{n}\left(L-l\right)}{4\left(F-F_{\tau }+\frac{F_{\tau }}{l}R\sin\alpha_{3}\right)} \end{array}\right.$$

**Figure 17:**
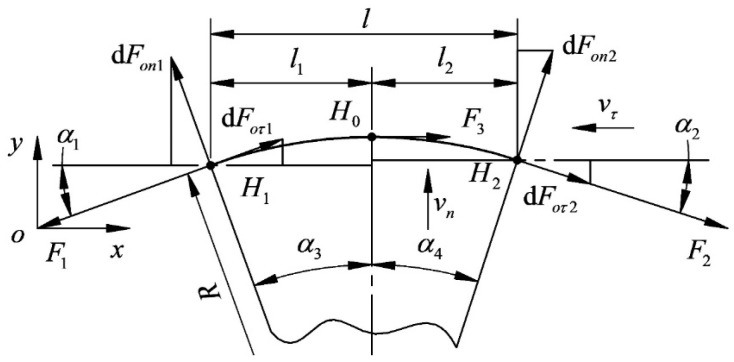
Force model of asymmetric circular wire bow.

Since the diamond wire tension $F_{3}$ at $H_{0}$ is in the horizontal direction, the straight line connecting the center of the arc and point $H_{0}$ must be perpendicular to the axis *x*, and the force model of the cutting segment can be constructed as shown in Figure 17.

Since the cutting segment of the wire bow is an arc, Equations (5) and (6) can be converted into

(16) $$\left\{ \begin{array}{l} dF_{on1}=\frac{\pi rK_{1}}{2}\cdot \frac{v_{n}\cos\alpha_{3}}{v_{\tau }-v_{n}\sin\alpha_{3}}\cdot Rd\alpha_{3}\\ dF_{o\tau 1}=2\pi rK_{2}\cdot \frac{v_{n}\cos\alpha_{3}}{v_{\tau }-v_{n}\sin\alpha_{3}}\cdot Rd\alpha_{3} \end{array}\right.$$

(17) $$\left\{ \begin{array}{l} dF_{on2}=\frac{\pi rK_{1}}{2}\cdot \frac{v_{n}\cos\alpha_{4}}{v_{\tau }+v_{n}\sin\alpha_{4}}\cdot Rd\alpha_{4}\\ dF_{o\tau 2}=2\pi rK_{2}\cdot \frac{v_{n}\cos\alpha_{4}}{v_{\tau }+v_{n}\sin\alpha_{4}}\cdot Rd\alpha_{4} \end{array}\right.$$

To obtain the cutting force in the theoretical wire bow model,

(18) $$\left\{ \begin{array}{l} F_{n}=R\int_{0}^{\alpha_{3}}\frac{\frac{\pi K_{1}}{2}\cdot rv_{n}\cos^{2}\alpha +2\pi K_{2}\cdot rv_{n}\cos\alpha \sin\alpha }{v_{\tau }-v_{n}\sin\alpha }d\alpha +R\int_{0}^{\alpha_{4}}\frac{\frac{\pi K_{1}}{2}\cdot rv_{n}\cos^{2}\alpha -2\pi K_{2}\cdot rv_{n}\cos\alpha \sin\alpha }{v_{\tau }+v_{n}\sin\alpha }d\alpha \\ F_{\tau }=R\int_{0}^{\alpha_{3}}\frac{-\frac{\pi K_{1}}{2}\cdot rv_{n}\cos\alpha \sin\alpha +2\pi K_{2}\cdot rv_{n}\cos^{2}\alpha }{v_{\tau }-v_{n}\sin\alpha }d\alpha +R\int_{0}^{\alpha_{4}}\frac{\frac{\pi K_{1}}{2}\cdot rv_{n}\cos\alpha \sin\alpha +2\pi K_{2}\cdot rv_{n}\cos^{2}\alpha }{v_{\tau }+v_{n}\sin\alpha }d\alpha \end{array}\right.$$

### 3.2. Calculation of Endpoint Tension

**Figure 18:**
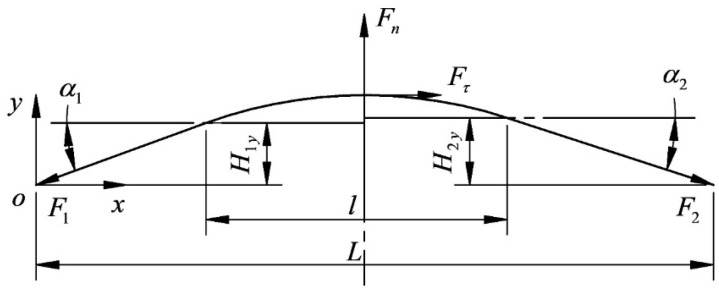
Force analysis diagram of wire bow.

Figure 18 shows the force analysis of the whole wire bow. Taking the wire speed to the left as an example, $F_{1}$ is the tension of the tight side (N), $F_{2}$ is the tension of the loose side (N), and $F_{1}$ and $F_{2}$ are collectively referred to as the endpoint tension on both sides.

Based on the force analysis of the wire bow, the expressions for $F_{1}$ and $F_{2}$ are obtained as follows:

(19) $$\left\{ \begin{array}{l} F_{1}=\frac{F_{\tau }}{\cos\alpha_{1}}+\frac{F_{n}\cos\alpha_{2}-F_{\tau }\tan\alpha_{1}\cos\alpha_{2}}{\sin\alpha_{1}\cos\alpha_{2}+\sin\alpha_{2}\cos\alpha_{1}}\\ F_{2}=\frac{F_{n}-F_{\tau }\tan\alpha_{1}}{\tan\alpha_{1}\cos\alpha_{2}+\sin\alpha_{2}} \end{array}\right.$$

## 4. Single-Wire Cutting Experiment and Analysis of Results

**Figure 19:**
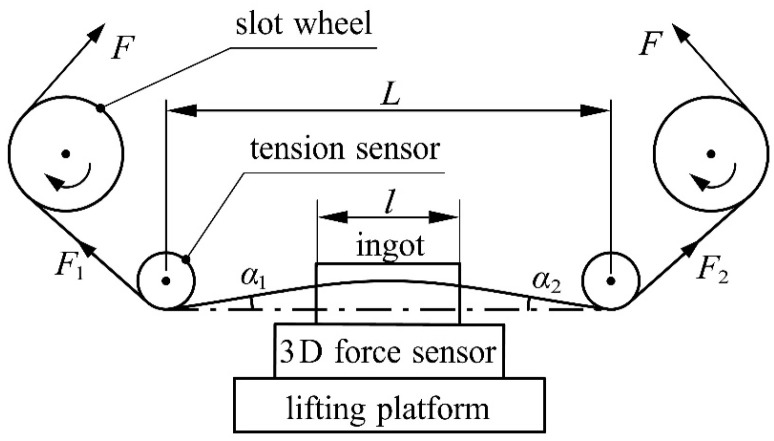
Schematic diagram of the experimental platform.

**Figure 20:**
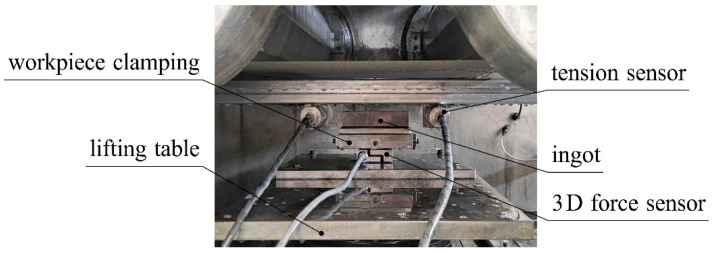
Physical diagram of the experimental platform.

To verify the wire bow model, it is necessary to obtain $F_{n}$, $F_{\tau }$, $H_{0y}$, $F_{1}$, $F_{2}$, $\alpha_{1}$ and $\alpha_{2}$ that correspond to different process parameters, where $\alpha_{1}$ and $\alpha_{2}$ are obtained indirectly through $F_{n}$ and $F_{\tau }$ as measured experimentally and combined with the finite element calculation. This experiment was carried out on a DX2260 multi-wire sawing machine. The same batch of HT250 was used as the experimental ingot, which was used after an aging treatment; the process parameters were controlled univariately; and the 3D force sensor was used to measure the normal cutting force and tangential cutting force of the ingot. Two symmetrically arranged tension sensors were used to obtain the endpoint tension of the wire bow. The experiment used a Viste VC60D three-dimensional force sensor to measure the cutting force of the material. The three-dimensional force sensor converts the measurement signal into a digital signal through an RS485 digital transmitter and transmits the signal in real time through a USB conversion connector connected to the computer. The special software on the computer was used to obtain and store the measurement data in real time. In the experiment, two JZHL-L1 single-pulley tension sensors were used to measure the end tension of the wire bow, and the measured data were converted into numerical signals by a double-channel transmitter, which was connected to the computer terminal through a USB adapter to realize data transmission. The maximum deflection of the wire bow was measured by a new method, and the measured wire bow parameters were compared with the theoretical calculation results to verify the wire bow model. Figure 19 is a schematic diagram of the experimental platform, and Figure 20 is a corresponding physical diagram.

**Figure 21:**
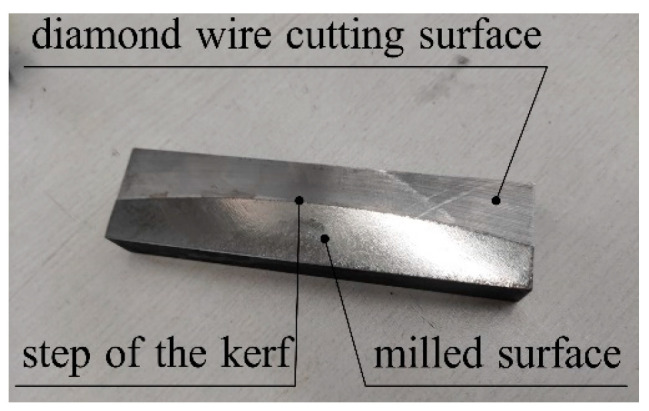
Physical diagram of the ingot after milling.

Unlike previous measurement methods used to determine wire bow deflection in cutting experiments, a new measuring method of interrupting the cutting is provided. In this experiment, after the cutting reached a stable stage, the cutting was stopped and the surface of the ingot was processed by milling to reveal the step of the kerf, which is the complete shape of the wire bow. Figure 21 shows the physical diagram of the ingot after milling.

**Figure 22:**
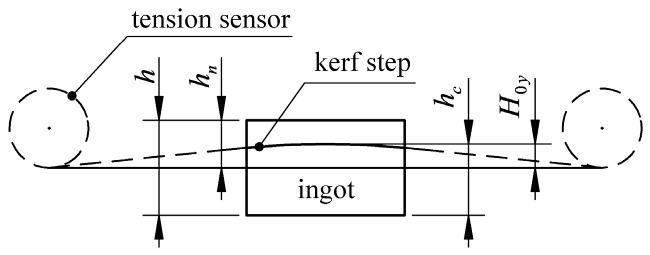
Schematic diagram of maximum deflection measurement.

Figure 22 is a schematic diagram of the maximum deflection measurement. The maximum deflection of the wire bow is obtained by measuring the profile of the wire bow, where $h_{n}$ in the figure is the feed height when the wire bow is stable. Corresponding to the moment when the endpoint tension $F_{1}$ and $F_{2}$ measured by the tension sensor are approximately constant, $h_{n}$ is read through the control interface of the machine.

Since the wire bow is a curve and has a maximum value, a vernier caliper is used to measure the kerf step multiple times, and based on the maximum value to obtain $h_{c}$, the maximum wire bow deflection $H_{0y}$ is

(20) $$H_{0y}=h_{c}-\left(h-h_{n}\right)$$

In the formula, $h_{c}$ is the measured height (mm), h is the height of the ingot (mm), and $h_{n}$ is the feed height (mm).

According to the investigation and the adjustable values of the experimental equipment, the experimental design of the wire bow model is shown in Table 3.

By selecting the experimental data of the variable group (feed speed $v_{n}$), the average value of $K_{1}$ and $K_{2}$ can be obtained:

(21) $$\left\{ \begin{array}{l} \bar{K_{1}}=282895\;\mathrm{MPa}\\ \bar{K_{2}}=61837\;\mathrm{MPa} \end{array}\right.$$

In the formula, $\bar{K_{1}}$ is the average normal comprehensive influence coefficient of HT250 (MPa) and $\bar{K_{2}}$ is the average tangential comprehensive influence coefficient of HT250 (MPa).

$\bar{K_{1}}$ and $\bar{K_{2}}$ are substituted into the theoretical model for calculation to obtain the corresponding wire bow parameters $F_{n}$, $F_{\tau }$, $H_{0y}$, $F_{1}$ and $F_{2}$, which are compared with the wire bow parameters measured by experiment.

$F_{nc}$ and $F_{\tau c}$ are values measured in the experiment corresponding to $F_{n}$ and $F_{\tau }$, $H_{0yc}$ is the value measured in the experiment corresponding to $H_{0y}$, $F_{1c}$ and $F_{2c}$ are values measured in the experiment corresponding to $F_{1}$ and $F_{2}$, and ΔF is set as the difference between $F_{1}$ and $F_{2}$ calculated using a theoretical calculation:

(22) $$\Delta F=F_{1}-F_{2}$$

In the formula, E is the difference between the endpoint tensions (N).

$\Delta F_{c}$ is set as the difference between $F_{1c}$ and $F_{2c}$ in the experiment.

(23) $$\Delta F_{c}=F_{1c}-F_{1c}$$

In the formula, $\Delta F_{c}$ is the difference between the endpoint tensions measured in the experiment (N).

**Figure 23:**
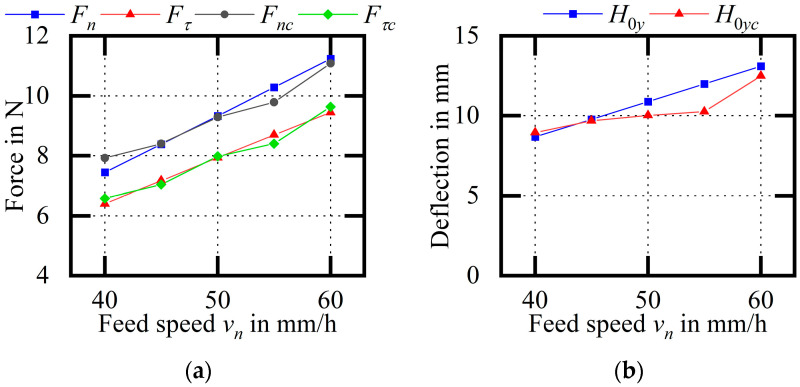

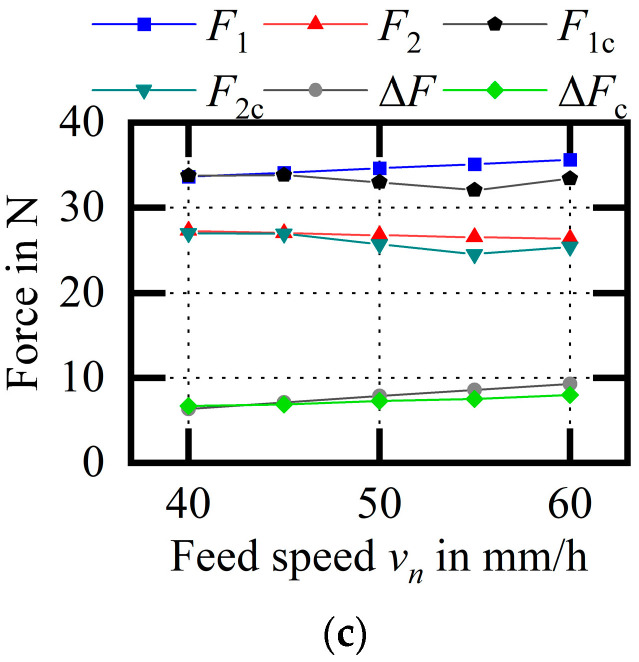
Experimental comparison of wire bow parameters (variable $v_{n}$). (**a**) Comparison of cutting forces; (**b**) comparison of $H_{0y}$ values; (**c**) comparison of endpoint tensions.

Figure 23 shows an experimental comparison of the wire bow parameters (variable $v_{n}$). According to an analysis of the wire bow parameters obtained via theory and the experiment, including the increase in feed speed, the normal and tangential cutting force, the maximum deflection, and the increase in the difference between endpoint tensions, the experimental results are similar to the theoretical calculation results, and the overall trend is the same. Among them, the average relative errors of $F_{n}$ and $F_{\tau }$ are 2.64% and 2.13%, respectively; the average relative error of $H_{0y}$ is 6.20%; and the average relative errors of $F_{1}$ and $F_{2}$ are 4.19% and 3.25%, respectively.

**Figure 24:**
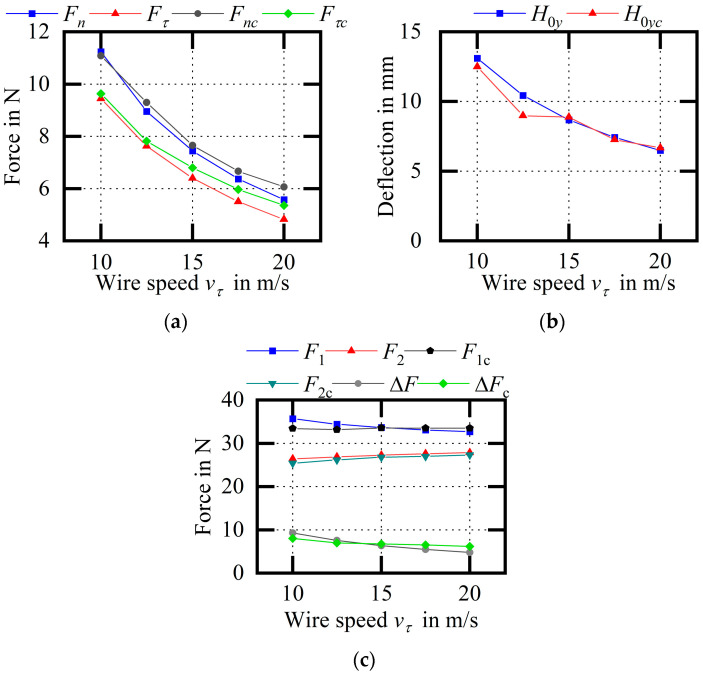
Experimental comparison of wire bow parameters (variable $v_{\tau }$). (**a**) Comparison of cutting forces; (**b**) comparison of $H_{0y}$ values; (**c**) comparison of endpoint tensions.

Figure 24 shows the experimental comparison of the wire bow parameters (variable $v_{\tau }$). Analysis of the data shows that with the increase in wire speed, the cutting force, the wire bow deflection, and the difference between endpoint tensions decrease. Among them, the average relative errors of $F_{n}$ and $F_{\tau }$ are 4.31% and 6.06%, respectively; the average relative error of $H_{0y}$ is 5.11%; and the average relative errors of $F_{1}$ and $F_{2}$ are 2.81% and 2.46%, respectively. The relative error between the experiment and the theoretical calculations in this group is small, which is beneficial for supporting the theoretical model.

**Figure 25:**
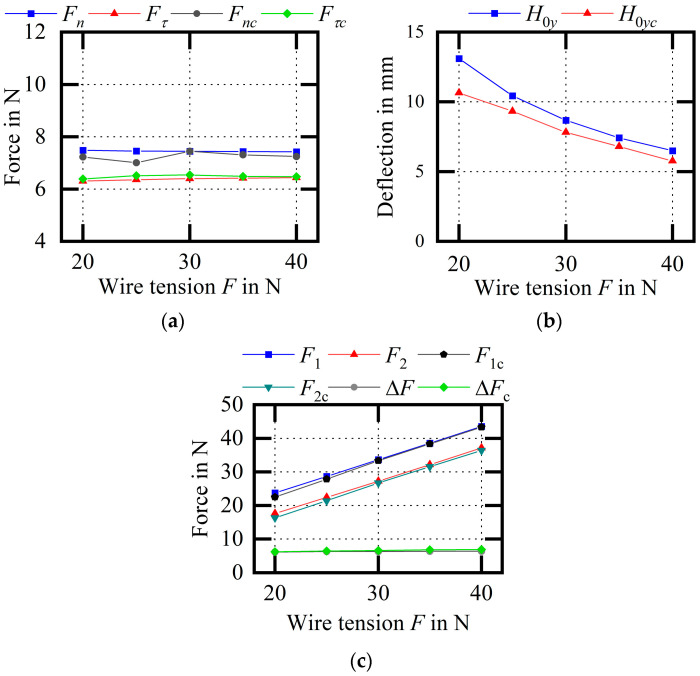
Experimental comparison of wire bow parameters (variable F). (**a**) Comparison of cutting forced; (**b**) comparison of $H_{0y}$ values; (**c**) comparison of endpoint tensions.

Figure 25 shows the experimental comparison of the wire bow parameters (variable F). As the wire tension increases, the cutting force remains unchanged, the wire bow deflection decreases, the endpoint tension increases, and the difference between the endpoint tensions is almost unchanged. Among them, the average relative errors of $F_{n}$ and $F_{\tau }$ are 2.72% and 1.53%, respectively; the average relative error of $H_{0y}$ is 11.77%; and the average relative errors of $F_{1}$ and $F_{2}$ are 2.00% and 3.56%, respectively. The experimental results of the maximum wire bow deflection are generally smaller than the theoretical calculation results, and the trend is consistent, which is beneficial for supporting the theoretical model.

**Figure 26:**
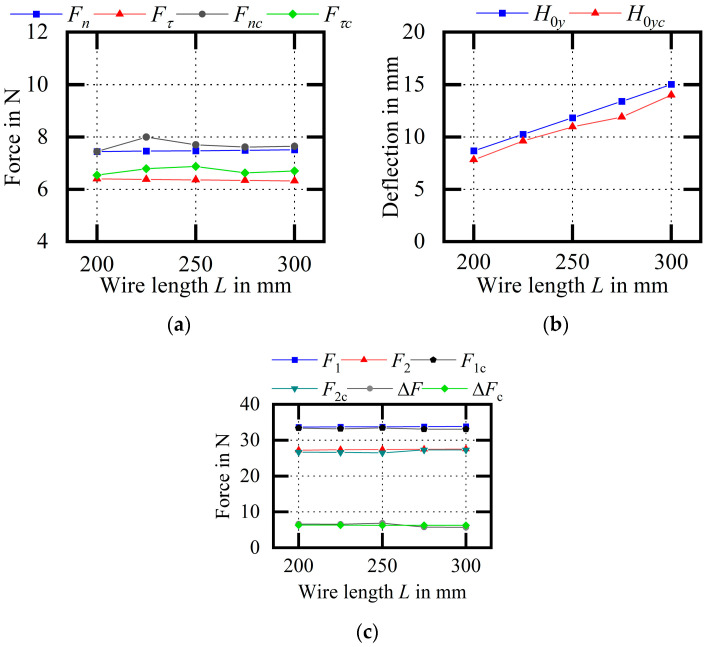
Experimental comparison of wire bow parameters (variable L). (**a**) Comparison of cutting forces; (**b**) comparison of $H_{0y}$ values; (**c**) comparison of endpoint tensions.

Figure 26 shows an experimental comparison of the wire bow parameters (variable L). With the increase in wire length, the cutting force remains unchanged, the deflection increases, and the endpoint tension and the difference remain unchanged. Among them, the average relative errors of $F_{n}$ and $F_{\tau }$ are 2.74% and 5.49%, respectively; the average relative error of $H_{0y}$ is 8.27%; and the average relative errors of $F_{1}$ and $F_{2}$ are 1.56% and 1.97%, respectively. Compared with the theoretical calculation, the error of the experimental result is smaller and the trend is consistent, which is mostly consistent with the calculation results from the theoretical model.

**Figure 27:**
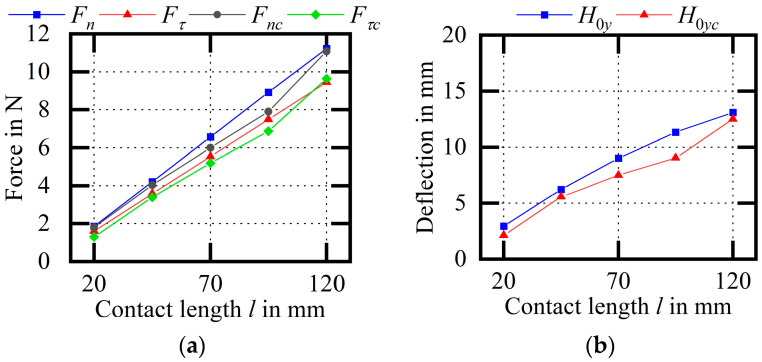

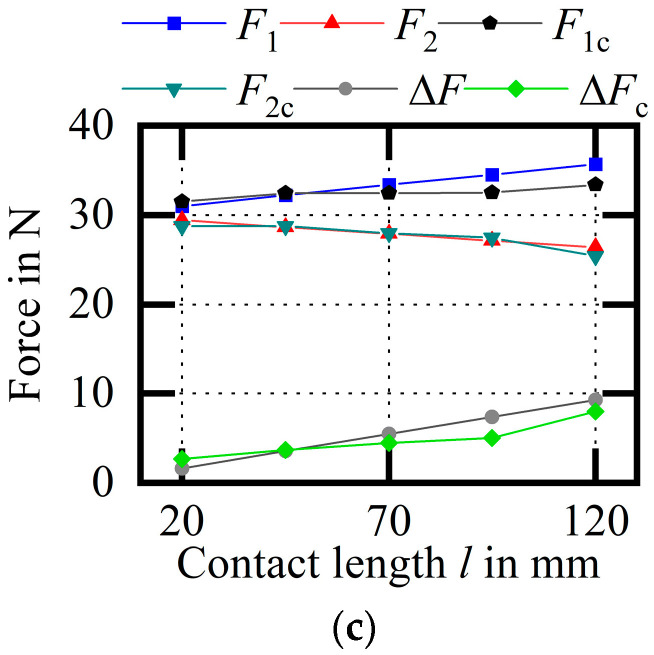
Experimental comparison of wire bow parameters (variable l). (**a**) Comparison of cutting forces; (**b**) comparison of $H_{0y}$ values; (**c**) comparison of endpoint tensions.

Figure 27 shows the experimental comparison of the wire bow parameters (variable l). As the contact length increases, the cutting force, the deflection and the difference between endpoint tensions increase. Among them, the average relative errors of $F_{n}$ and $F_{\tau }$ are 6.41% and 5.29%, respectively; the average relative error of $H_{0y}$ is 13.08%; and the average relative errors of $F_{1}$ and $F_{2}$ are 3.48% and 1.51%, respectively. When the contact length is relatively small, the denominator of the relative error is small; thus, the data at l=20 mm are discarded. The measured maximum deflection of the wire bow is generally smaller than the theoretical calculation, the trend is consistent, and other wire bow parameters are relatively accurate.

**Figure 28:**
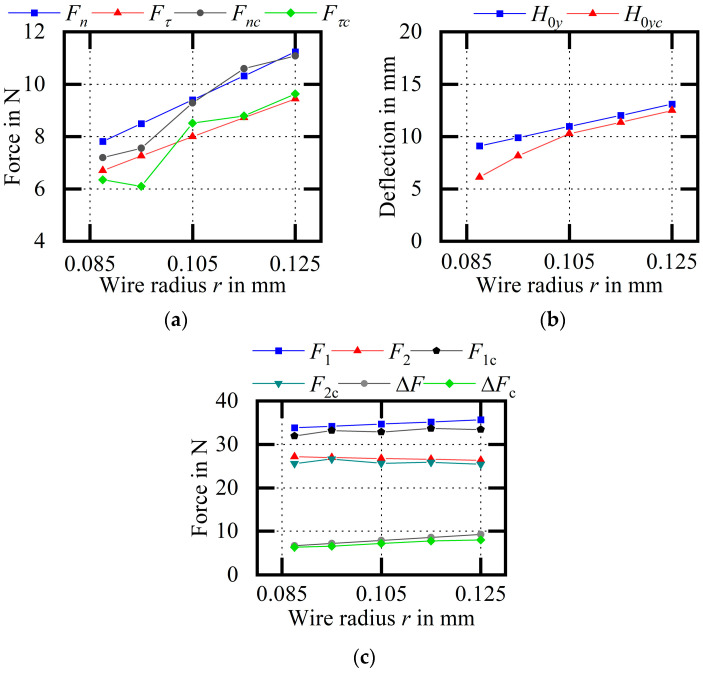
Experimental comparison of wire bow parameters (variable r). (**a**) Comparison of cutting forces; (**b**) comparison of $H_{0y}$ values; (**c**) comparison of endpoint tensions.

Figure 28 shows an experimental comparison of the wire bow parameters (variable r). As the wire radius increases, the cutting force, the deflection, and the difference between endpoint tensions increase. For the three groups of experimental results with wire radii of 0.105 mm, 0.115 mm and 0.125 mm, the average relative errors of $F_{n}$ and $F_{\tau }$ are 1.75% and 3.09%, respectively; the average relative error of $H_{0y}$ is 5.49%; and the average relative errors of $F_{1}$ and $F_{2}$ are 5.25% and 3.43%, respectively.

The single-variable control method was used in the analytical model and experiment, and the relationship between each process parameter and wire bow parameters was obtained, as shown in Table 4.

- The normal comprehensive influence coefficient $K_{1}$ mainly affects the change in $F_{n}$. As $K_{1}$ increases, the maximum deflection $H_{0y}$ of the wire bow increases, the deflection angles on both sides increase, and the endpoint tension tends to be stable.
- The tangential comprehensive influence coefficients $K_{2}$ and $K_{1}$ are related to each other and have the same increasing and decreasing relationship, but by analyzing $K_{2}$ independently from the perspective of the calculation, it can be found to have a greater effect on $F_{\tau }$. When the increases in $K_{2}$, $F_{n}$, and $H_{0y}$ remain the same, the deflection angle on the tight side $\alpha_{1}$ decreases, the deflection angle on the loose side $\alpha_{2}$ increases, and the difference between the endpoint tensions increases; $F_{\tau }$ is the main factor that causes the asymmetric offset of the wire bow, and $K_{2}$ plays a leading role in $F_{\tau }$. When the ingot is difficult to process, the differences between the deflection angles and endpoint tensions of the wire bow increase, the asymmetry of the wire bow is enhanced, the difference in the endpoint tensions on a single diamond wire increases, and the cutting stability is weakened.
- The feed speed $v_{n}$ has an important effect on the change in the wire bow, and the cutting force $F_{n}$ and $F_{\tau }$ increase with the increase in $v_{n}$. When the wire bow reaches the stable stage, the $H_{0y}$ increases, the deflection angles on both sides increase, and the difference between endpoint tensions on both sides increases; at this time, the asymmetry of the wire bow is enhanced and the cutting stability is weakened.
- With the increase in the wire speed $v_{\tau }$, the cutting ability of the diamond wire increases; $F_{n}$, $F_{\tau }$ and $H_{0y}$ all decrease; the deflection angles on both sides decrease; the difference between the endpoint tensions decreases; the asymmetry of the wire bow is weakened; and the cutting stability is enhanced.
- The wire tension F does not affect the cutting force $F_{n}$ and $F_{\tau }$, but as F increases, the maximum deflection of the wire bow $H_{0y}$ decreases, the deflection angles on both sides decrease, the endpoint tension increases linearly, the difference between the endpoint tensions remains unchanged, and the asymmetry of the wire bow is not affected.
- The total length of the wire L mainly affects the maximum deflection of the wire bow $H_{0y}$, and $H_{0y}$ increases with the increase in L.
- The contact length l has an effect on each parameter; with the increase in l, the cutting force $F_{n}$ and $F_{\tau }$, the maximum deflection of the wire bow $H_{0y}$, the deflection angles on both sides, the difference between the endpoint tensions, and the asymmetry of the wire bow are all enhanced, but the cutting stability is weakened.
- As the wire radius r increases, $F_{n}$, $F_{\tau }$, $H_{0y}$, $\alpha_{1}$, and $\alpha_{2}$ all increase, the difference between endpoint tensions increases, the asymmetry of the wire bow is enhanced and the cutting stability is weakened.

Based on the comprehensive analysis of the calculation results, it can be determined that F and L will not lead to changes in the cutting force, the cutting force cannot be used as a standard to judge the cutting state of the diamond wire, and the process parameters $v_{n}$, $v_{\tau }$, F, L, l, and r will have an effect on $H_{0y}$; therefore, $H_{0y}$ can be used to indicate the cutting state corresponding to different process parameters. The model takes into account the asymmetry of the wire bow and solves the magnitude of two endpoint tensions on both sides. There is always a difference between the two endpoint tensions of the wire bow. The diamond wire tension has a safe range of variation, and the smaller the change, the more stable it is; that is, the smaller the difference between the endpoint tensions on both sides, the more stable it is. Therefore, the difference can be expressed as the stability of the wire bow, and the model can provide a tension range for the selection of a diamond wire.

$K_{1}$ and $K_{2}$ are mainly affected by material of ingot, the physical properties of diamond wire, and cutting fluid, which reflect the difficulty of cutting a certain ingot; with the increase in processing difficulty, $K_{1}$ and $K_{2}$ increase at the same time.

Compared with the experimental data, the wire bow parameters calculated using the theoretical model have generally smaller errors, and the trend is the same. For the whole experiment, the errors of $F_{n}$ and $F_{\tau }$ were 3.43% and 3.93%, respectively; the error of $H_{0y}$ was 8.32%; and the errors of $F_{1}$ and $F_{2}$ were 3.21% and 2.70%, respectively. The above results show the accuracy of the theoretical model, establishing that the main advantage of the wire bow model is that the wire bow parameters such as $H_{0y}$, $F_{1}$, and $F_{2}$ can be accurately calculated after the $K_{1}$ and $K_{2}$ is determined by trial cutting the ingot. $K_{1}$ and $K_{2}$ are mainly affected by the material’s properties, the physical properties of the diamond line, and the cutting fluid, reflecting the difficulty of processing a certain material; that is, with the increase in processing difficulty, $K_{1}$ and $K_{2}$ increase at the same time.

Due to the bending of the diamond wire, the feed height required to cut through the ingot is the height of the ingot added to the maximum deflection of the wire bow:

(24) $$h_{n}=h+H_{0y}$$

In the formula, h is the height of the ingot (mm) and $h_{n}$ is the feed height when the ingot is cut (mm).

The maximum deflection calculated by the model can be used to determine the feed height $h_{n}$, the unnecessary time to eliminate the wire bow deflection can be reduced by the accurate calculation of $h_{n}$, and the production efficiency can be improved.

The time needed to completely cut the ingot can be expressed as

(25) $$t=\frac{h+H_{0y}}{v_{n}}$$

The maximum deflection of the wire bow $H_{0y}$ is affected by the process parameters $v_{n}$, $v_{\tau }$, F, L, l, and r, and $H_{0y}$ reflects the quality of the processing. The time when the ingot of the same height is cut through with different process parameters can be expressed as the cutting efficiency; when h and $v_{n}$ remain unchanged, the larger the value of $H_{0y}$, the greater the bending degree of the diamond wire, and the more difficult it is to process the ingot with these process parameters, the worse the cutting efficiency. Only increasing $v_{n}$ can obviously improve the cutting efficiency, but the effect of $v_{n}$ on $H_{0y}$ needs to be considered, that is, the effect of $v_{n}$ on the cutting quality; therefore, the theoretical model can be used for the calculation, the process parameters can be changed to adjust $H_{0y}$, the relationship between the cutting efficiency and cutting quality can be measured, and a comprehensive reference can be provided for the selection of the process parameters.

## 5. Conclusions

The asymmetric arc hypothesis is put forward, which shows that the cutting segment of the wire bow can be described as an asymmetric arc. Error analysis between the fitted arc deflection and the wire bow deflection based on a theoretical derivation and finite element calculation was carried out, which proved the rationality of the asymmetric arc hypothesis. Based on the hypothesis and the sensitivity analysis of the boundary conditions, the theoretical wire bow model was established, and a single-wire cutting experiment was designed to verify it.

The model systematically describes the mathematical relationship between the process parameters and the wire bow parameters. In addition to calculating the cutting force, the wire bow deflection, the deflection angles, and the endpoint tensions corresponding to different process parameters can be accurately obtained. The relevant conclusions are as follows:

- The model explains the effect of process parameters $v_{n}$, $v_{\tau }$, F, L, l, and r on the bending deformation of a diamond wire from the theoretical level. The maximum wire bow deflection calculated theoretically can be used as a standard to represent the bending state of the diamond wire and provide a calculation basis for the selection of process parameters.
- Using $H_{0y}$ to calculate the feed height $h_{n}$ required for processing reduces the time needed to eliminate wire bow deflection and improve cutting efficiency.
- The cutting efficiency is expressed by time t, and combined with the effect of $H_{0y}$ on the cutting quality, a mathematical model for measuring the relationship between the cutting quality and cutting efficiency is given, which provides a theoretical reference for the matching scheme of process parameters.
- The model takes the asymmetry of the wire bow into account, and the endpoint tensions of the wire bow $F_{1}$ and $F_{2}$ can be calculated. By using the wire bow model, the mathematical formula of the tension change in the cutting segment is given, showing that there is a difference between the endpoint tensions on both sides, the difference of which can provide a reference for the cutting stability and a tension range for the selection of the diamond wire.

## Figures and Tables

**Table 1:** Selection of working condition parameters for wire bow fitting.

Parameters	F/N	$F_{n}$/N	$F_{\tau }$/N	L/mm	l/mm	r/mm
F	30~50	6.5	6.5	400	175	0.1
$F_{n}$	40	3~10	6.5	400	175	0.1
$F_{\tau }$	40	6.5	3~10	400	175	0.1
L	40	6.5	6.5	300~500	175	0.1
l	40	6.5	6.5	400	100~250	0.1
r	40	6.5	6.5	400	175	0.075~0.125

**Table 2:** Parameters of maximum fitting error within the range of working condition parameters.

F/N	$F_{n}$/N	$F_{\tau }$/N	L/mm	l/mm	r/mm
30	10	10	300	250	0.125

**Table 3:** Experimental design of the wire bow model.

Set of Variables	$v_{n}$/(mm/h)	$v_{\tau }$/(m/s)	F/N	L/mm	l/mm	r/mm
Feed speed $v_{n}$	40	10.0	30	200	120	0.1250
	45					
	50					
	55					
	60					
Wire speed $v_{\tau }$	60	12.5	30	200	120	0.1250
		15.0				
		17.5				
		20.0				
Wire tension F	60	15.0	20	200	120	0.1250
			25			
			30			
			35			
			40			
Wire length L	60	15.0	30	225	120	0.1250
				250		
				275		
				300		
Contact length l	60	10.0	30	200	20	0.1250
					45	
					70	
					95	
Wire radius r	60	10.0	30	200	120	0.0875
						0.0950
						0.1050
						0.1150

**Table 4:** Qualitative results of analytical model and experiment, symbols: ↗—value increases, ↘—value decreases, Co.—value remains unchanged, $F_{n}$—normal cutting force of cutting segment, $F_{\tau }$—tangential cutting force of cutting segment, $H_{0y}$—maximum deflection of wire bow, $\alpha_{1}$—deflection angle on tight side of the wire bow, $\alpha_{2}$—deflection angle on loose side of the wire bow, $F_{1}$—endpoint tension on tight side of wire bow, $F_{2}$—endpoint tension on loose side of wire bow.

Parameters	$F_{n}$	$F_{\tau }$	$H_{0y}$	$\alpha_{1}$	$\alpha_{2}$	$F_{1}$	$F_{2}$
Normal comprehensive influence coefficient $K_{1}$↗	↗	Co.	↗	↗	↗	Co.	Co.
Tangential comprehensive influence coefficient $K_{2}$↗	Co.	↗	Co.	↘	↗	↗	↘
Feed speed $v_{n}$↗	↗	↗	↗	↗	↗	↗	↘
Wire speed $v_{\tau }$↗	↘	↘	↘	↘	↘	↘	↗
Wire tension F↗	Co.	Co.	↘	↘	↘	↗	↗
Wire length L↗	Co.	Co.	↗	Co.	Co.	Co.	Co.
Contact length l↗	↗	↗	↗	↗	↗	↗	↘
Wire radius r↗	↗	↗	↗	↗	↗	↗	↘

## Data Availability

Not applicable.
